# Circulating dipeptidyl peptidase-3 at admission is associated with circulatory failure, acute kidney injury and death in severely ill burn patients

**DOI:** 10.1186/s13054-020-02888-5

**Published:** 2020-04-22

**Authors:** François Dépret, Juliette Amzallag, Adrien Pollina, Laure Fayolle-Pivot, Maxime Coutrot, Maïté Chaussard, Karine Santos, Oliver Hartmann, Marion Jully, Alexandre Fratani, Haikel Oueslati, Alexandru Cupaciu, Mourad Benyamina, Lucie Guillemet, Benjamin Deniau, Alexandre Mebazaa, Etienne Gayat, Boris Farny, Julien Textoris, Matthieu Legrand, for the PRONOBURN group

**Affiliations:** 1 Department of Anesthesiology and Critical Care and Burn Unit, AP-HP, GH St-Louis-Lariboisière, Paris, France; 2 University Paris Diderot, Paris, France; 3 UMR INSERM 942, Institut National de la Santé et de la Recherche Médicale (INSERM), F-CRIN INICRCT network, Paris, France; 4 Department of Anesthesiology and Critical Care, Burn Center Pierre Colson, Hospices Civils de Lyon, Edouard Herriot Hospital, Lyon, France; 5 4TEEN4 Pharmaceuticals GmbH, Hennigsdorf, Germany; 6 EA 7426 Pathophysiology of Injury-induced Immunosuppression, University of Lyon1-Hospices Civils de Lyon-bioMérieux, Hôpital Edouard Herriot, Lyon, France; 7 Department of Anesthesia and Perioperative Care, UCSF Medical Center, University of California, 500 Parnassus Avenue MUE416, Box 0648, San Francisco, CA 94143 USA

**Keywords:** Dipeptidyl peptidase-3, Burn patients, Mortality, Acute kidney injury, Biomarkers

## Abstract

**Background:**

Dipeptidyl peptidase-3 (DPP3) is a metallopeptidase which cleaves bioactive peptides, notably angiotensin II, and is involved in inflammation regulation. DPP3 has been proposed to be a myocardial depressant factor and to be involved in circulatory failure in acute illnesses, possibly due to angiotensin II cleavage. In this study, we evaluated the association between plasmatic DPP3 level and outcome (mortality and hemodynamic failure) in severely ill burn patients.

**Methods:**

In this biomarker analysis of a prospective cohort study, we included severely ill adult burn patients in two tertiary burn intensive care units. DPP3 was measured at admission (DPP3_admin_) and 3 days after. The primary endpoint was 90-day mortality. Secondary endpoints were hemodynamic failure and acute kidney injury (AKI).

**Results:**

One hundred and eleven consecutive patients were enrolled. The median age was 48 (32.5–63) years, with a median total body surface area burned of 35% (25–53.5) and Abbreviated Burn Severity Index (ABSI) of 8 (7–11). Ninety-day mortality was 32%. The median DPP3_admin_ was significantly higher in non-survivors versus survivors (53.3 ng/mL [IQR 28.8–103.5] versus 27.1 ng/mL [IQR 19.4–38.9]; *p* < 0.0001). Patients with a sustained elevated DPP3 had an increased risk of death compared to patients with high DPP3_admin_ but decreased levels on day 3. Patients with circulatory failure had higher DPP3_admin_ (39.2 ng/mL [IQR 25.9–76.1] versus 28.4 ng/mL [IQR 19.8–39.6]; *p* = 0.001) as well as patients with AKI (49.7 ng/mL [IQR 30.3–87.3] versus 27.6 ng/mL [IQR 19.4–41.4]; *p* = 0.001). DPP3_admin_ added prognostic value on top of ABSI (added chi^2^ 12.2, *p* = 0.0005), Sequential Organ Failure Assessment (SOFA) score at admission (added chi^2^ 4.9, *p* = 0.0268), and plasma lactate at admission (added chi^2^ 6.9, *p* = 0.0086) to predict circulatory failure within the first 48 h.

**Conclusions:**

Plasma DPP3 concentration at admission was associated with an increased risk of death, circulatory failure, and AKI in severely burned patients. Whether DPP3 plasma levels could identify patients who would respond to alternative hemodynamic support strategies, such as intravenous angiotensin II, should be explored.

## Background

Severe burn injury is associated with an early and profound hypovolemia followed by an intense systemic inflammatory response. Hemodynamic management, including fluid resuscitation, has long been recognized as the cornerstone of the early management and hemodynamic resuscitation of severely burned patients [1–3]. However, a systemic inflammatory response may be associated with distributive shock and/or acute myocardial dysfunction [4]. Dipeptidyl peptidase-3 (DPP3), also named enkephalinase B or red cell angiotensinase, is a predominantly intracellular, ubiquitously expressed, zinc-dependent metallopeptidase involved in the metabolism of peptides [5] implicated in many different pathways (e.g., blood pressure regulation, inflammation). DPP3 cleaves bioactive peptides, notably angiotensin II, enkephalins, and endomorphins [6–8]. We hypothesize that cleavage of angiotensin II by DPP3 may promote vasodilatation and circulatory failure. Severe burn patients are at high risk of developing vasodilatory shock with systemic inflammatory response after the early phase of hypovolemic shock. The main objective of this study was therefore to assess the association between DPP3 at admission (DPP3_admin_) and day 3 (DPP3_Day3_) with 90-day mortality in severely burned patients. The secondary objective was to evaluate the association between DPP3 and organ dysfunction (i.e., circulatory failure and acute kidney injury (AKI)).

## Methods

### Study design and population

We conducted a double-center cohort study in the Burn Unit of Saint Louis Hospital (Assistance Publique Hôpitaux de Paris), Paris, and in the Burn Unit of Edouard Herriot Hospital, Lyon, France. The study was approved by our local ethic committee (PRONOBURN study, comité de protection des personnes IV, St-Louis Hospital; Institutional Review Board 00003835, protocol 2013/17NICB). All patients admitted to our intensive care burn units (ICBU) between April 2014 and April 2016 and meeting the inclusion criteria were included. Inclusion criteria were the following:

- A total body surface area [TBSA] burned > 15%
- Admission in the ICBU within the 72 h following burn injury
- No decision to withdraw life support
- Available plasma sample at admission

### Outcome

The endpoints were 90-day mortality, circulatory failure in the first 48 h, and AKI.

### Measurements

The following data have been collected: sex, age, body mass index (BMI), TBSA, full-thickness body surface area (BSA) burned, mechanism of injury and patients’ characteristics, Abbreviated Burn Severity Index (ABSI) [9], Unit Burn Standard (UBS) [10], Sequential Organ Failure Assessment (SOFA) score [11], 28- and 90-day mortality, and AKI. Patients were resuscitated according to the Intensive Care Burn Unit resuscitation protocol [12]. A transthoracic or transesophageal echocardiography was performed at admission of patients according to the decision of the physician in charge. When performed, left ventricular ejection fraction (LVEF) was visually evaluated and systolic cardiac dysfunction was defined by a LVEF < 50% [13]. Circulatory failure was defined as a need for hemodynamic support with inotrope and/or a vasopressor (i.e., dobutamine, epinephrine, or norepinephrine) despite appropriate fluid resuscitation in the first 48 h. We choose this time frame to identify circulatory failure related to burn injury (as opposed to sepsis or surgical procedures which occur later during the course of burn injuries). AKI was defined and staged according to the Kidney Disease: Improving Global Outcomes (KDIGO) criteria [14] during the first 7 days post admission. Admission serum creatinine e (Screat_admin_) was used as baseline Screat.

Venous blood samples were collected at admission and at day 3 in tubes containing ethylene-diamine-tetra-acetic acid. After centrifugation, plasma was kept frozen at − 80 °C until assayed. DPP3 was measured using the recently developed DPP3 luminescence immunoassay [15].

### Statistical analysis

Values are expressed as medians and interquartile ranges (IQR) or counts and percentages as appropriate. Group comparisons of continuous variables were performed using Kruskal-Wallis test. Categorical data were compared using Pearson’s chi-squared test for count data. DPP3 data was log-transformed. Cox proportional-hazards regression was used to analyze the effect of risk factors on survival in uni- and multivariable analyses, and logistic regression was used for dichotomous endpoints. In both cases, to demonstrate independence from other variables, the added value of DPP3 on top of these was evaluated based on the likelihood ratio chi-squared test for nested models. The concordance index (C index or AUC) is given as an effect measure for uni- and multivariable models. For multivariable models, a bootstrap-corrected version of the C index is given. For continuous variables, hazard ratios (HR) or odds ratios (OR), as appropriate, were standardized to describe the HR/OR for a change of one IQR. HR (Cox regression) are used if time-to-event data is available; OR (logistic regression) are used if endpoints have event data (yes/no) only. Survival curves plotted by the Kaplan-Meier method were used for illustrative purposes. For dichotomous endpoints, receiver operating characteristic (ROC) curves were constructed for illustration. All statistical tests were 2-tailed and a two-sided *p*-value of 0.05 was considered for significance. The statistical analyses were performed using R version 3.4.3 (http://www.r-project.org, library rms, Hmisc, ROCR) and Statistical Package for the Social Sciences (SPSS) version 22.0 (SPSS Inc., Chicago, Illinois, USA).

## Results

### Study population

From April 2014 to April 2016, 208 consecutive patients met the inclusion criteria; 55 patients had missing plasma at admission and were not included in the final analysis, resulting in 111 patients that were analyzed. The characteristics of the patients included in this study are summarized in Table 1. The median age was 48 (32.5–63) years, with a median TBSA of 35% (25–53.5) and median ABSI of 8 (7–11). All patients had a DPP3_admin_ measurement and 79 patients (71%) had DPP3_Day3_ (10 patients died before day 3 and 22 patients had missing measurements at day 3).

**Table 1:** Patients characteristics

Patient’s characteristics	Total, ***N*** = 111	90-day survivors, ***N*** = 75	90-day non-survivors, ***N*** = 36	***p***
Sex, male—*n* (%)	71 (64)	51 (68)	20 (56)	0.2858
Age—year	48 [32.5–63]	42 [29–58]	56.5 [42–79]	0.0013
BMI—kg/m^2^	25.2 [22.9–28.7]	25.1 [23–28.3]	25.7 [22.4–29.1]	0.9673
**Medical history**				
CIC—*n* (%)	3 (2.7)	2 (3)	1 (3)	1.0000
COPD—*n* (%)	3 (2.7)	2 (3)	1 (3)	1.0000
CKD—*n* (*n*)	5 (4.5)	1 (1)	4 (11)	0.0374
Chronic HBP—*n* (%)	25 (22.5)	12 (16)	13 (36)	0.0277
Psychiatric—*n* (%)	34 (30.6)	22 (29)	12 (33)	0.6668
**Burn characteristics**				
TBSA—%	35 [25–53.5]	32 [22–45]	57 [31–70]	< 0.0001
Deep burn BSA—%	21 [10–40]	17 [7–30]	42 [15–61]	0.0001
Inhalation injury—*n* (%)	54 (48.6)	28 (37)	26 (72)	0.0012
**Characteristics during hospitalization**				
Mechanic ventilation—n (%)	82 (73.9)	52 (69)	30 (83)	0.1799
DPP3_admin_ (ng/mL)	30.6 [22.4–53.6]	27.1 [19.4–40.2]	53.3 [29.5–104]	< 0.0001
DPP3_day3_ (ng/mL)	17.3 [11.8–25.2]	14.1 [11.5–20.6]	22.1 [16.6–30.8]	0.0102
Screat—μmol/L	72.5 [56.5–92]	70 [54.8–81.3]	90.5 [67.3–138.3]	0.0003
Lactate—mmol/L	3.5 [2.0–5.7]	2.7 [1.7–4.6]	5.2 [3.5–8]	< 0.0001
Bilirubin—mmol/L	14.0 [9.3–21.3]	12.9 [9–19.3]	18 [10.9–25.9]	0.0945
Platelet—G/L	250 [185–304]	236 [183–277]	279 [180–372]	0.3840
Length of hospitalization—days	90 [35.5–90]	41 [26–61]	18 [2–32.5]	< 0.0001
RRT—*n* (%)	24 (21.6)	5 (7)	28 (78)	< 0.0001
**Severity scores**				
SOFA	4 [1–7]	2 [0–4]	6.5 [3.3–9.8]	< 0.0001
ABSI	8 [7–11]	8 [6–9]	11 [9–13]	< 0.0001
SAPS2	33 [23–47]	28 [20–42]	47 [33–62]	< 0.0001
UBS	100 [52.5–166]	84 [45–132]	184 [86–249]	< 0.0001
**Hemodynamic on admission**				
Echocardiography, *n* (%)	59 (53)	33 (44)	26 (72)	0.0078
Systolic cardiac dysfunction, *n* (%)	10 (9)	2 (3)	8 (22)	0.0163
Circulatory failure, *n* (%)	53 (48)	24 (32)	29 (81)	< 0.0001
MAP in mmHg	79 [70–95]	84 [73–97]	73 [64–85]	0.0104
Volume of crystalloids at day 1	8250 [3700–15,000]	6700 [3300–12,800]	13,400 [6430–18,380]	0.0018
Volume of crystalloids at day 2	3000 [1000–5650]	2500 [1000–5150]	4000 [2000–7500]	0.1078

*BMI* body mass index, *CIC* chronic ischemic cardiopathy, *COPD* chronic obstructive pulmonary disease, *CKD* chronic kidney disease, *HBP* high blood pressure, *TBSA* total burn surface area, *ECMO* extracorporeal membrane oxygenation, *Screat* serum creatinine at admission, *RRT* renal replacement therapy, *SOFA score* simplified organ failure assessment, *ABSI* Abbreviated Burn Severity Index, *UBS* Unit Burn Standard, SAPS 2 The Simplified Acute Physiology Score

### DPP3_admin_ and 90-day mortality

**Fig. 1:**
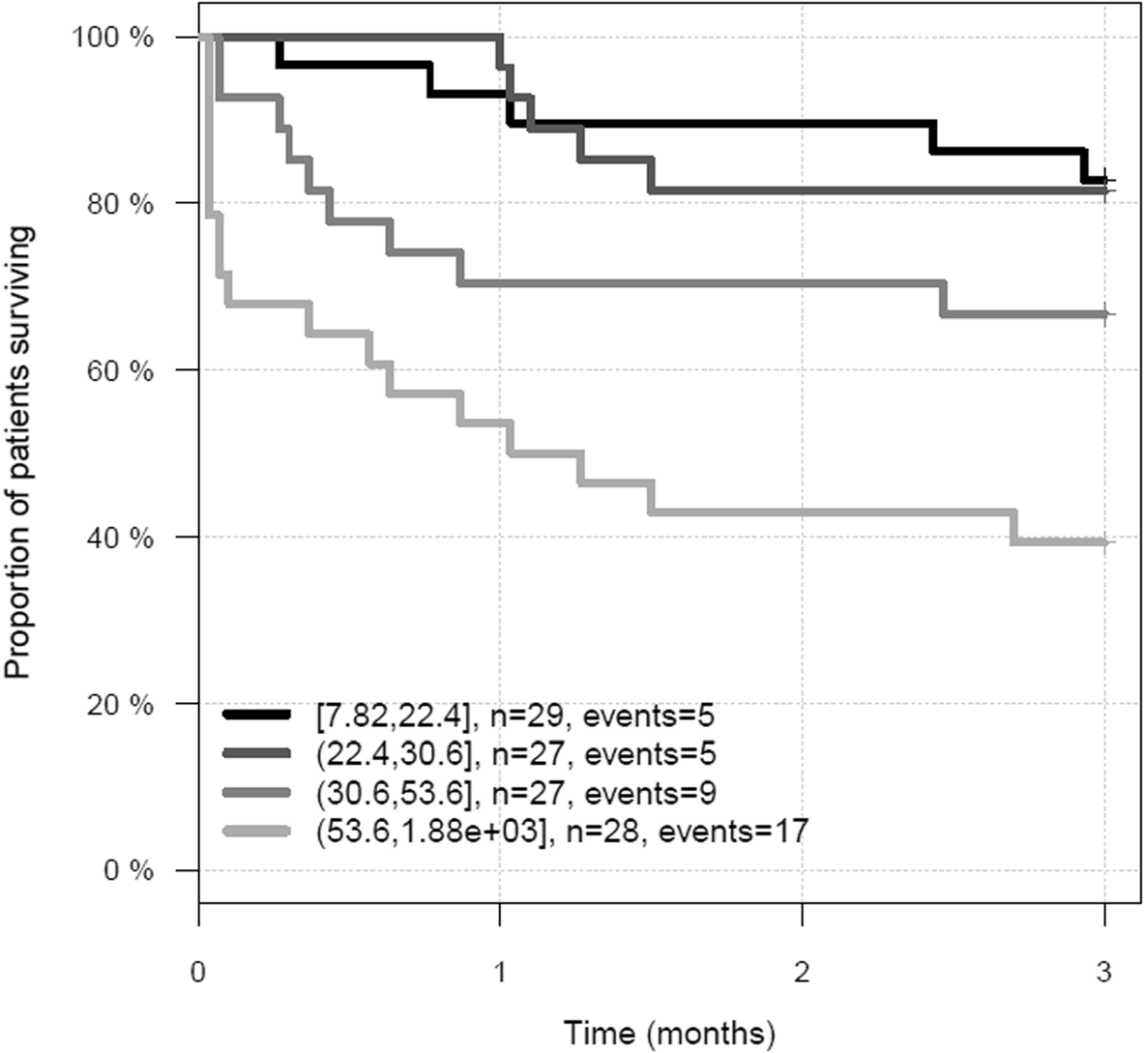
Represents a survival Kaplan-Meier curve depending on DPP3_admin_ quartiles (legend gives quartile ranges for DPP3 in nanograms/milliliter)

**Fig. 2:**
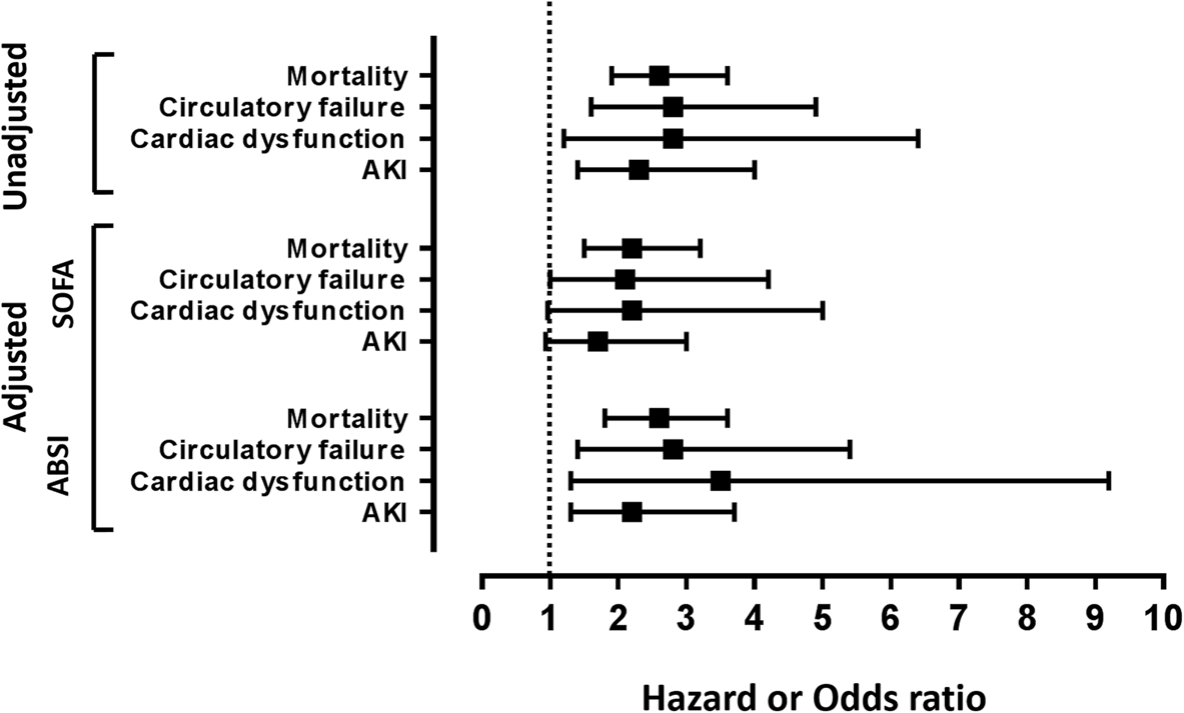
Represents unadjusted and adjusted (i.e., on Sequential Organ Failure Assessment-SOFA-OFA score or Abbreviated Burn Severity Index-ABSI) hazard ratio (HR) and/or odds ratio (OR) of DPP3 _admin_ value and outcomes (i.e., mortality, cardiac dysfunction, circulatory failure and acute kidney injury, AKI, respectively). Mortality *n* = 111/36 events, HR not adjusted HR = 2.6 (1.9–3.6); adjusted on SOFA score, HR = 2.2 (1.5–3.2); and adjusted on ABSI, HR = 2.6 (1.8–3.6), respectively. Circulatory failure, *n* = 111/53 events, OR not adjusted: OR = 2.8 (1.6–4.9); adjusted on SOFA score, HR = 2.1 (1*.*0–4.2) and adjusted on ABSI, HR = 2.8 (1.4–5.4), respectively. Cardiac dysfunction, *n* = 59/10 events, OR not adjusted: OR = 2.8 (1.2–6.4); adjusted on SOFA score, HR = 2.2 (0.96–5.0) and adjusted on ABSI, HR = 3.5 (1.3–9.2), respectively. Acute kidney injury (AKI) *n* = 111/35 events, OR not adjusted: OR = 2.3 (1.4–4.0); adjusted on SOFA score, HR = 1*.*7 (0.93–3.0); and adjusted on ABSI, HR = 2*.2* (1.3–3.7), respectively

**Fig. 3:**
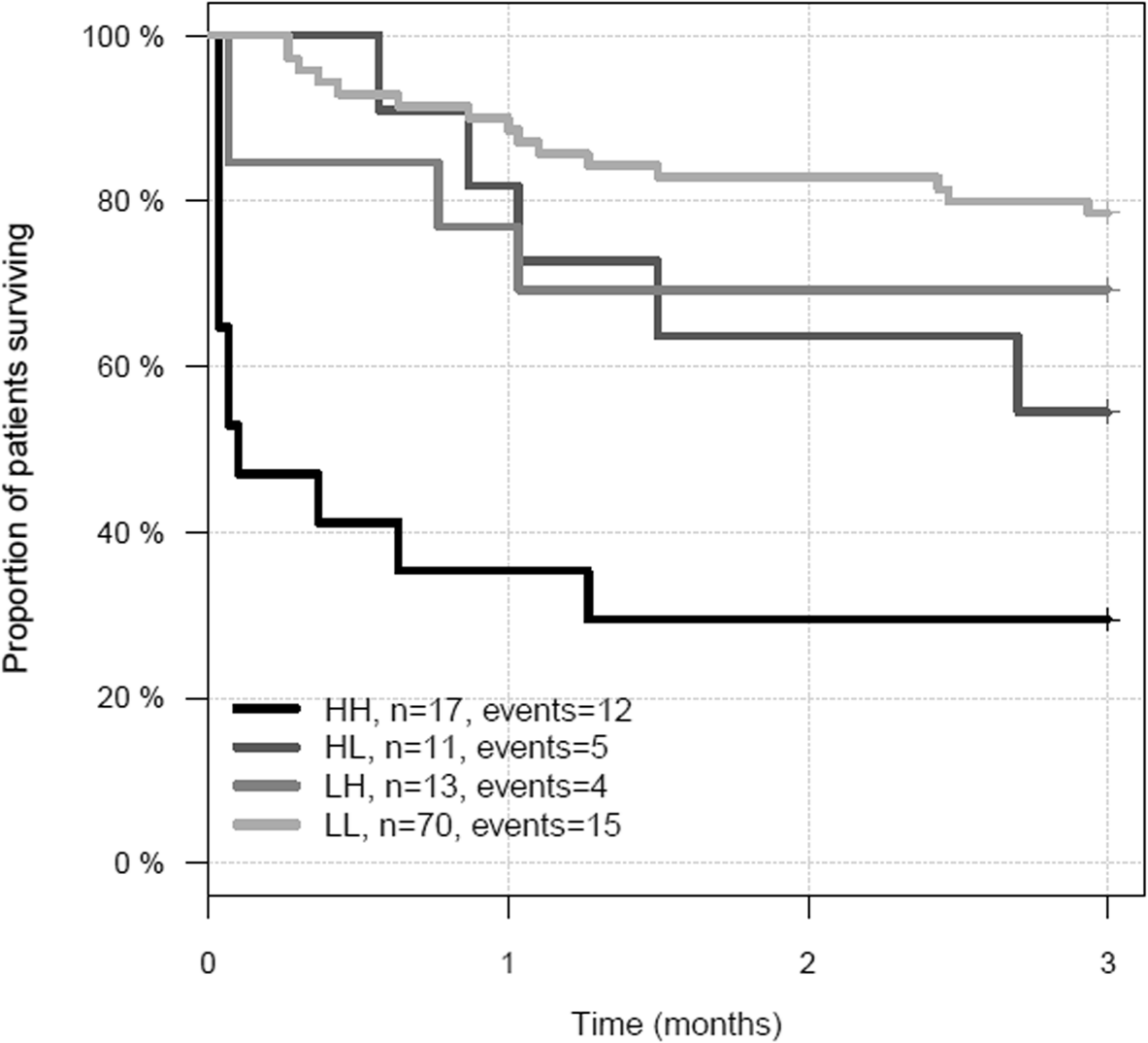
Represents an illustration of the added value of DPP3_day 3_ using a cut point of 53.65 ng/mL at admission and day 3. Patients without DPP3 data at day 3 were left in their subgroup assigned to on day 1. High at admission and high at day 3 (HH): patients above 53.65 ng/mL at admission and at day 3; high at admission and low at day 3 (HL): patients above 53.65 ng/mL at admission and below 53.65 ng/mL at day 3; low at admission and high at day 3 low high (LH): patients below 53.65 ng/mL at admission and above 53.65 ng/mL at day 3; low at admission and low at day 3 (LL): Patients below 53.65 ng/mL at admission and on day 3. Cut point identified is the third quartile (53.65 ng/mL)

Thirty-six (32%) patients died before day 90. Median DPP3_admin_ was significantly higher in non-survivors versus survivors (53.3 ng/mL [IQR 28.8–103.5] versus 27.1 ng/mL [IQR 19.4–38.9]; *p* < 0.0001). We observed a stepwise increase in mortality among quartile groups of DPP3_admin_, the patients in the highest quartile having the highest mortality (Fig. 1). There was no interaction between DPP3 value and TBSA (*p* = 0.7132) (Supplementary Figure 1). The C index of DPP3_admin_ for 90-day mortality was 0.734 (0.653–0.815, *p* < 0.0001, standardized HR 2.6 (1.9–3.6)). DPP3_admin_ added prognostic value on top of ABSI (added chi^2^ 24.5, *p* < 0.0001), SOFA score at admission (SOFA_admin_, added chi^2^ 15.4, *p* < 0.0001), and lactate at admission (added chi^2^ 11.7, *p* = 0.0006) to predict 90-day mortality (Fig. 2). Furthermore, adding DPP3_Day3_ to DPP3_admin_ provided added value to predict 90-day mortality (added chi^2^ 5.6, *p* = 0.018; missing data at day 3 replaced with admission data). Patients with a high DPP3_admin_ that decreased at day 3 had a better prognosis than patients with high DPP3_admin_ and sustained DPP3_Day3_ (Fig. 3).

### DPP3 and circulatory failure

**Fig. 4:**
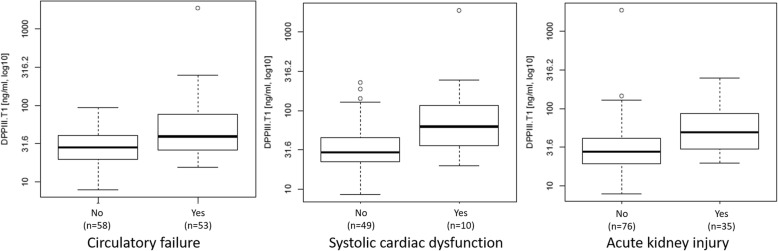
Represents median DPP3 _admin_ between patients with and without circulatory failure in the first 48 h (left panel), between patients with or without systolic cardiac dysfunction at admission (middle panel), and between patients with or without acute kidney injury (right panel)

Fifty-three (48%) patients had circulatory failure during the first 48 h (44 patients received norepinephrine, five patients received dobutamine + norepinephrine, 4 patients received epinephrine). DPP3_admin_ was significantly higher in patients with circulatory failure compared to patients without (39.2 ng/mL [IQR 25.9–76.1] versus 28.4 ng/mL [IQR 19.8–39.6]; *p* = 0.001) (Fig. 4 left panel). DPP3_admin_ was associated with circulatory failure with an AUC of 0.680 (0.581–0.778, *p* < 0.0001, standardized OR 2.8 (1.6–4.9)). DPP3_admin_ provided value on top of ABSI (added chi^2^ 12.2, *p* = 0.0005), SOFA score at admission (SOFA_admin_, added chi^2^ 4.9, *p* = 0.0268), and lactate at admission (added chi^2^ 6.9, *p* = 0.0086) to predict hemodynamic support in the first 48 h. There was no correlation between DPP3 and the volume administered on day 1 (*r* = 0.17, *p* = 0.07).

Fifty-nine patients (53%) had an echocardiography performed at admission. Among them, 10 (17%) patients had a systolic cardiac dysfunction. DPP3_admin_ was significantly higher in patients with systolic cardiac dysfunction compared to patients without (62.4 ng/mL [IQR 40.4–112.3] versus 29.3 ng/mL [IQR 22.4–45.1]; *p* < 0.0122) (Fig. 4 middle panel). The area under the ROC curve for DPP3_admin_ to predict systolic cardiac dysfunction was 0.753 (95%CI 0.582–0.925, *p* = 0.0054).

### DPP3 and acute kidney injury

Thirty-five (32%) patients developed AKI during the first 7 days. DPP3_admin_ was significantly higher in patients with AKI compared to patients without (49.7 ng/mL [IQR 30.3–87.3] versus 27.6 ng/mL [IQR 19.4–41.4]; *p* = 0.001) (Fig. 4 right panel). DPP3_admin_ was associated with AKI with an AUC of 0.735 (0.641–0.829, *p* = 0.0005, standardized OR 2.3 (1.4–4.0)). DPP3_admin_ added value on top of ABSI (added chi^2^ 9.4, *p* = 0.0022), SOFA score at admission (SOFA_admin_, added chi^2^ 14.3, *p* = 0.0002), but not on top of creatinine at admission (added chi^2^ 0.3, *p* = 0.5954) to predict AKI.

## Discussion

In this biomarker analysis of a prospective cohort, we observed that DPP3_admin_ was strongly associated with 90-day mortality, circulatory failure, and AKI in severely burned patients. Furthermore, adding DPP3_admin_ to SOFA_admin_, lactate_admin_, or ABSI outperformed these prognostic factors to predict 90-day mortality. Serial measurements of DPP3 have improved the prediction of outcome compared to DPP3_admin_ alone.

While the prognosis of burn patients has improved, the mortality of most severe patients remains high with many patients dying from circulatory failure and multiple organ failure [16, 17]. Initial hemodynamic management has long been considered critical in the treatment and prognosis of burn patients [18]. Burn injury is characterized by an initial hypodynamic state with low cardiac output due to hypovolemia followed by a hyperdynamic state with high cardiac output and low vascular resistance developing 12 to 24 h after the injury [1]. The severity of the distributive shock and occurrence of cardiac dysfunction may, however, vary greatly between patients. The association between DPP3 levels, circulatory failure, and AKI is consistent with the current understanding of AKI in the critically ill, associating hemodynamic factors and inflammation/immune response [19, 20]. These results might also be expected in patients developing systemic inflammatory response from different causes (e.g., sepsis, post-cardiopulmonary bypass, post-cardiac arrest, pancreatitis), and it should be further explored.

In the current study, DPP3 was strongly associated with mortality and hemodynamic failure, even after adjustment for classic markers of severity and prognosis. Recently, Deniau et al. observed an association between high plasmatic levels of DPP3 and high mortality and organ dysfunction in severe heart failure patients. Furthermore, I.V. administration of DPP3 rapidly deteriorated cardiac contraction in mice [21]. In an ancillary study of the OptimaCC study, Takagi et al. showed that high circulating DPP3 was associated with low cardiac index, refractory shock, and high mortality in patients with cardiogenic shock [22].

The results of the present study have several potential implications for future research. First, the identification of patients with high plasma DPP3 may trigger cardiac function assessment. Second, high DPP3 levels at admission may help to select candidate patients for alternative vasopressor therapies, especially for infusion of angiotensin II [23, 24]. Angiotensin II has been found to be downregulated in some forms of septic shock associated with poor prognosis [25]. DPP3 cleaves angiotensin II and may, therefore, play a role in vasoplegic shock by reducing angiotensin II levels. Since angiotensin II is not easy to measure in clinical practice, DPP3 may represent a potential candidate biomarker for selecting patients most likely to respond to angiotensin II infusion. Third, pharmacological inhibition of DPP3 by a specific antibody has been shown to promptly restore and sustain cardiac contraction in mice [21] and might be a therapeutic option in burn patients with high DPP3. All these strategies are hypothesis and require exploration and validation in well-designed prospective human studies.

Our study has several limitations. First, the observational design of the present study does not allow us to conclude on the causality between DPP3 and mortality or organ dysfunction. Second, the study contains a relatively low number of patients, even though this is one of the largest cohort studies among critically ill burn patients with sufficient power to identify an association between the biomarker levels and outcomes*.* Thirdly, factors influencing DPP3 metabolism are unknown and will need further exploration in critically ill burn patients. Finally, only half of our patients had an echocardiography at admission, limiting the interpretation of the association between DPP3 levels and cardiac dysfunction.

## Conclusion

Plasma DPP3 concentration at admission was associated with an increased risk of death, circulatory failure, and AKI in severely burned patients. Whether DPP3 plasma levels could identify patients who would respond to alternative hemodynamic support strategies, such as intravenous angiotensin II, should be explored.

## Supplementary information

**Additional file 1: Figure S1.** represents interaction between Total Body Surface Area (TBSA) and DPP3_admin (TIF 43 kb)_

**Additional file 2.** Supplementary data Table 1. Patients characteristics according to TBSA (Total burn surface area).

## Data Availability

The datasets used and/or analyzed during the current study are available from the corresponding author on reasonable request.

## Abbreviations

DPP3: Dipeptidyl peptidase-3
AKI: Acute kidney injury
ICBU: Intensive care burn unit
TBSA: Total body surface area burn
BMI: Body mass index
BSA: Body surface area
ABSI: Abbreviated Burn Severity Index
UBS: Unit Burn Standard
SOFA: Sequential Organ Failure Assessment
LVEF: Left ventricular ejection fraction
KDIGO: Kidney Disease: Improving Global Outcomes
IQR: Interquartile range
HR: Hazard ratio
OR: Odds ratio
ROC: Receiver operator characteristics
