# Supplementary figures and images for: Circulating dipeptidyl peptidase-3 at admission is associated with circulatory failure, acute kidney injury and death in severely ill burn patients
Source: Crit Care. 2020 Apr 22;24:168. doi: 10.1186/s13054-020-02888-5 (PMC7178561; doi:10.1186/s13054-020-02888-5)

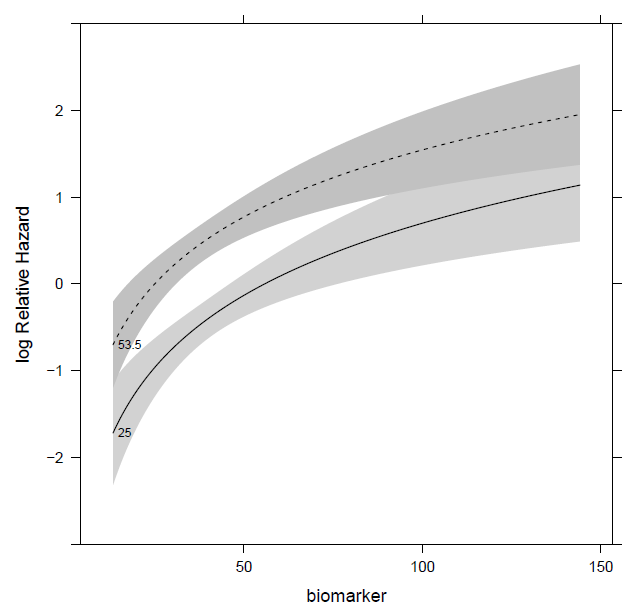

Supplement: Supplementary file 1 — Additional file 1: Figure S1. represents interaction between Total Body Surface Area (TBSA) and DPP3admin (TIF 43 kb) [file 13054_2020_2888_MOESM1_ESM.tif]
