# Supplementary material for: Circulating dipeptidyl peptidase-3 at admission is associated with circulatory failure, acute kidney injury and death in severely ill burn patients
Source: Crit Care. 2020 Apr 22;24:168. doi: 10.1186/s13054-020-02888-5 (PMC7178561; doi:10.1186/s13054-020-02888-5)
Supplement: Supplementary file 2 — Additional file 2. Supplementary data Table 1. Patients characteristics according to TBSA (Total burn surface area). [file 13054_2020_2888_MOESM2_ESM.docx]

**Supplementary data Table 1 - Patient characteristics:**

| **Patient’s characteristics** | **Total** | **TBSA <35%** | **TBSA ≥35%** | **p** |
| --- | --- | --- | --- | --- |
|  | **N = 111** | **N = 55** | **N = 56** |  |
| Sex Male – n (%) | 71 (64) | 40 (71.4) | 31 (56.4) | 0,1456 |
| Age – Year | 48 [32.5-63] | 46 [34.5-66.5] | 48 [28-57] | 0.4520 |
| **Medical history** |  |  |  |  |
| CIC – n (%) | 3 (2.7) | 2 (3.6) | 1 (1.8) | 1.0000 |
| COPD – n (%) | 3 (2.7) | 2 (3.6) | 1 (1.8) | 1.0000 |
| CKD – n (n) | 5 (4.5) | 3 (5.4) | 2 (3.6) | 1.0000 |
| Chronic HBP – n (%) | 25 (22.5) | 14 (25) | 11 (20) | 0.6867 |
| Psychiatric – n (%) | 34 (30.6) | 16 (28.6) | 18 (32.7) | 0.7879 |
| **Burn characteristics** |  |  |  |  |
| TBSA - % | 35 [25-53.5] | 25 [20-30.25] | 54 [45-65] | ND |
| Deep burn BSA – % | 21 [10-40] | 10 [5-18] | 40 [27.5-58] | ND |
| Inhalation injury – n (%) | 54 (48.6) | 19 (33.9) | 35 (63.6) | 0.0033 |
| **Characteristics during hospitalization** |  |  |  |  |
| Mechanic ventilation – n (%) | 82 (73.9) | 30 (53.6) | 52 (94.5) | <0.0001 |
| Screat - µmol/l | 72.5 [56.5-92] | 72 [60.5-91] | 73 [55.5-109.5] | 0.7649 |
| Lactate – mmol/l | 3.5 [2.0-5.7] | 2.6 [1.7-4.6] | 4.3 [2.7-6.6] | 0.0043 |
| Bilirubin – mmol/l | 14.0 [9.3-21.3] | 12.0 [9.0-19.2] | 15.7 [10.8-22.6] | 0.1705 |
| Platelet – G/l | 249.5 [185-303.5] | 230 [175.5-281.5] | 262 [200.5-321.5] | 0.1215 |
| Length of hospitalization – Days* | 90 [35.5-90] | 90 [90-90] | 90 [22.5-90] | 0.0101 |
| Death within 90 days | 36 (32.4) | 11 (19.6) | 25 (45.5) | 0.0069 |
| RRT – n (%) | 24 (21.6) | 7 (12.5) | 17 (30.9) | 0.0424 |
| **Severity scores** |  |  |  |  |
| SOFA | 4 [1-7] | 2 [0-4.25] | 6 [3-8] | <0.0001 |
| ABSI | 8 [7-11] | 7 [6-8] | 11 [9-12.5] | <0.0001 |
| SAPS2 | 33 [23-47] | 27 [18.75-38.25] | 39 [28-49.5] | 0.0027 |
| UBS | 100 [52.5-166] | 54.5 [37-81] | 167 [130-240] | <0.0001 |
| **Biomarker** |  |  |  |  |
| DPP3_admin_ | 30.6 [22.4-53.6] | 30.0 [20.2-45.8] | 33.4 [24.9-59.6] | 0.1721 |
|  |  |  |  |  |
|  |  |  |  |  |
| CIC: chronic ischemic cardiopathy; COPD: Chronic obstructive pulmonary disease; CKD: Chronic kidney disease; HBP: high blood pressure; TBSA: Total burn surface area; Screat: serum Creatinine at admission; RRT: Renal replacement therapy; SOFA score: simplified organ failure assessment; ABSI: Abbreviated burn severity index; UBS: unit burn standard; SAPS 2: The Simplified Acute Physiology Score 2 ; DPP3_admin_: Dipeptidyl Peptidase 3 at admission  *we set LOS = 90 days for all survivors | | | | |
